# Autophagy Induced by Intracellular Infection of *Propionibacterium acnes*

**DOI:** 10.1371/journal.pone.0156298

**Published:** 2016-05-24

**Authors:** Teruko Nakamura, Asuka Furukawa, Keisuke Uchida, Tomohisa Ogawa, Tomoki Tamura, Daisuke Sakonishi, Yuriko Wada, Yoshimi Suzuki, Yuki Ishige, Junko Minami, Takumi Akashi, Yoshinobu Eishi

**Affiliations:** 1 Department of Human Pathology, Graduate School and Faculty of Medicine, Tokyo Medical and Dental University, Tokyo 113–8510, Japan; 2 Division of Surgical Pathology, Tokyo Medical and Dental University Hospital, Tokyo 113–8510, Japan; 3 Department of Clinical Engineering, School of Health Sciences, Tokyo University of Technology, Tokyo 144–8650, Japan; Aarhus University, DENMARK

## Abstract

**Background:**

Sarcoidosis is caused by Th1-type immune responses to unknown agents, and is linked to the infectious agent *Propionibacterium acnes*. Many strains of *P*. *acnes* isolated from sarcoid lesions cause intracellular infection and autophagy may contribute to the pathogenesis of sarcoidosis. We examined whether *P*. *acnes* induces autophagy.

**Methods:**

Three cell lines from macrophages (Raw264.7), mesenchymal cells (MEF), and epithelial cells (HeLa) were infected by viable or heat-killed *P*. *acnes* (clinical isolate from sarcoid lymph node) at a multiplicity of infection (MOI) of 100 or 1000 for 1 h. Extracellular bacteria were killed by washing and culturing infected cells with antibiotics. Samples were examined by colony assay, electron-microscopy, and fluorescence-microscopy with anti-LC3 and anti-LAMP1 antibodies. Autophagy-deficient (Atg5^-/-^) MEF cells were also used.

**Results:**

Small and large (≥5 μm in diameter) LC3-positive vacuoles containing few or many *P*. *acnes* cells (LC3-positive *P*. *acnes*) were frequently found in the three cell lines when infected by viable *P*. *acnes* at MOI 1000. LC3-positive large vacuoles were mostly LAMP1-positive. A few small LC3-positive/LAMP1-negative vacuoles were consistently observed in some infected cells for 24 h postinfection. The number of LC3-positive *P*. *acnes* was decreased at MOI 100 and completely abolished when heat-killed *P*. *acnes* was used. LC3-positive *P*. *acnes* was not found in autophagy-deficient Atg5^-/-^ cells where the rate of infection was 25.3 and 17.6 times greater than that in wild-type Atg5^+/+^ cells at 48 h postinfection at MOI 100 and 1000, respectively. Electron-microscopic examination revealed bacterial cells surrounded mostly by a single-membrane including the large vacuoles and sometimes a double or multi-layered membrane, with occasional undigested bacterial cells in ruptured late endosomes or in the cytoplasm.

**Conclusion:**

Autophagy was induced by intracellular *P*. *acnes* infection and contributed to intracellular bacterial killing as an additional host defense mechanism to endocytosis or phagocytosis.

## Introduction

Sarcoidosis, a systemic granulomatous disease that may occur in genetically susceptible subjects exposed to an environmental agent, has clinical similarities with infectious granulomatous diseases, suggesting that sarcoidosis has a microbial etiology [1]. Activated T cells and macrophages comprise the inflammatory response of sarcoidosis [2], and the pattern of cytokine production in the lungs is consistent with the helper T-cell type 1 (Th1) immune response that is triggered by an unidentified antigen(s) [3].

*Propionibacterium acnes*, commensal bacteria on human skin and mucosal surfaces, may cause sarcoidosis. *P*. *acnes* is the only microorganism isolated to date from bacterial cultures of sarcoid lesions [4,5]. Studies using quantitative polymerase chain reaction have detected *P*. *acnes* DNA in sarcoid lymph nodes [6,7], and studies using in situ hybridization have revealed *P*. *acnes* in sarcoid granulomas [8]. Further, immunohistochemistry studies with monoclonal antibodies for *P*. *acnes* identified *P*. *acnes*-positive cells in granulomas from lung and lymph node tissues obtained from patients with sarcoidosis [9]. Stimulation of sarcoidosis patients by viable *P*. *acnes* cells [10] or their bacterial components [11–13] leads to increased Th1 immune responses.

Many strains of *P*. *acnes* isolated from sarcoid lesions can infect epithelial cells [14], leading to the activation of nuclear factor (NF)-*κ*B in both a nucleotide-binding oligomerization domain-containing protein 1 (NOD1)- and NOD2-dependent manner [15]. Recently, Fischer et al. reported the survival and persistence of *P*. *acnes* in macrophages [16], and Negi et al. [9] found latent *P*. *acnes* infection in sinus macrophages of the lymph nodes and many *P*. *acnes* in some macrophages or granuloma cells at the site of sarcoid inflammatory lesions. Such evidence for intracellular persistence and possible intracellular proliferation of *P*. *acnes* suggests that allergic Th1 immune responses in sarcoidosis patients are triggered by intracellular proliferation of persistent *P*. *acnes* at the site of latent infection.

Autophagy, a group of degradation pathways that deliver cytoplasmic constituents to lysosomes, is one of several mechanisms that defend against intracellular pathogens. Although group A streptococcus [17], *Listeria monocytogenes* [18], and *Shigella flexneri* [19] can escape from phagocytic vacuoles, they are quickly absorbed by autophagosomes decorated with the autophagy marker LC3. The autophagy machinery also target bacteria restricted to the vacuoles, such as *Mycobacteria tuberculosis* [20] and *Salmonella enterica* serovar Typhimurium [21]. Thus, while the mechanisms that induce autophagy in response to intracellular bacteria remain unclear, autophagy appears to be part of a broad host response to intracellular bacteria and not induced by specific bacteria.

This study examined whether a cell-invasive strain of *P*. *acnes* isolated from a sarcoid lymph node can induce autophagy after intracellular infection of three cell lines: macrophages, mesenchymal cells, and epithelial cells.

## Materials & Methods

### Reagents and Antibodies

Rabbit polyclonal antibody against LC3 was purchased from MBL (PM036; Nagoya, Japan), and rat monoclonal antibody against LAMP1 (ab25245) was obtained from Abcam (Cambridge, MA, USA). Mouse monoclonal antibody against LAMP1 (sc-20011) was purchased from Santa Cruz Biotechnology (Dallas, TX, USA). Biotinylated anti-rabbit immunoglobulin antibody (E0432; DAKO, Glostrup, Denmark), FITC-conjugated streptavidin (F0422; DAKO), Alexa647 conjugated anti-rat immunoglobulin antibody (A-21247; Molecular Probes, Waltham, MA, USA), and Alexa647 conjugated anti-mouse immunoglobulin antibody (115-606-146; Jackson ImmunoResearch Laboratories, West Grove, PA, USA) were used for detection.

### *P*. *acnes* strain

A cell-invasive strain of *P*. *acnes*, a clinical isolate obtained from a lymph node affected by sarcoidosis [22] was used in this study. *P*. *acnes* were cultured on Gifu Anaerobic (GAM) broth (Nissui, Tokyo, Japan) for 3 d at 37°C under anaerobic conditions. For heat inactivation, *P*. *acnes* was incubated at 100°C for 10 min.

### Cell culture

A mouse macrophage cell line, Raw264.7 (American Type Culture Collection, Manassas, VA, USA), was routinely cultured in RPMI-1640 with 10% heat-inactivated fetal bovine serum (FBS) and 50 μg/ml gentamycin. Mouse embryonic fibroblasts (MEF cells) of autophagy-deficient type (Atg5^-/-^) and of wild-type (Atg5^+/+^) [23], and the human cervix adenocarcinoma cell line HeLa (American Type Culture Collection) were routinely cultured in Dulbecco's Modified Eagle Medium (DMEM) with 10% heat-inactivated FBS, 40 U/ml penicillin, and 100 μg/ml streptomycin. They were cultured at 37°C in an incubator with a humidified atmosphere of 5% CO_2_.

### Infection

For *P*. *acnes* infection, the bacteria were harvested from 3-d cultures in stationary phase and washed twice with phosphate buffered saline (PBS). Bacterial density was adjusted to optical density at 600 nm (OD_600_) = 2. *P*. *acnes* were added to the cell culture (2×10^5^ Raw264.7 cells, 5×10^4^ MEF cells, and 1×10^5^ HeLa cells) on cover-glasses (Matsunami Glass Ind., Ltd., Osaka, Japan) coated with 0.1% gelatin/PBS in a 24-well culture plate at a 1:100 or 1:1000 multiplicity of infection (MOI) without antibiotics. After 1 h, infected cells were washed three times with PBS, and then 10% FBS/DMEM with antibiotics (400 U/ml penicillin G and 1 mg/ml streptomycin) was added to kill extracellular bacteria.

### Immunofluorescence confocal microscopy

Infected cells were fixed in 4% paraformaldehyde for 15 min and permeabilized with 0.1% Triton X-100 solution in PBS for 10 min, followed by incubation in 3% bovine serum albumin/PBS + 10 mg/ml RNaseA (Wako Pure Chemical Industries Ltd., Osaka, Japan) overnight at 4°C. The cells were incubated with primary antibodies for 60 min and secondary antibodies for 30 min, followed by three 5 min-washes in PBS. Finally, cells were stained with propidium iodide nucleic acid stain (0.5 μg/ml, Invitrogen-Molecular Probes) for 40 min and mounted with Fluor Save^TM^ Reagent (Calbiochem, Billerica, MA, USA). All steps were performed at room temperature. The cells were observed under a fluorescence laser-scanning microscope (FV1200, Olympus, Tokyo, Japan). The degree of autophagy induction was determined by the frequency (%) of cells with at least one LC3-positive vacuole containing *P*. *acnes* in 100 to 150 randomly-counted *P*. *acnes*-infected cells.

### Electron microscopy

For electron microscopy, cells infected with *P*. *acnes* on chamber slides were fixed with 2.5% glutaraldehyde in 0.1 M phosphate buffer (pH 7.4) for 2 h. Conventional electron microscopy was performed as follows. After washing five times with 0.1 M phosphate buffer, the cells were post-fixed with 2% osmium tetroxide and 0.5% potassium ferrocyanide in the same buffer for 1 h, and washed again with 0.1 M phosphate buffer. After dehydration, the cells were embedded in Epon 812 (TAAB Laboratories Equipment Ltd.). Ultrathin sections were stained by uranyl acetate plus lead citrate and observed with an H7700 electron microscope (Hitachi, Tokyo, Japan).

### Colony assay for intracellular viable *P*. *acnes*

Colony assay for intracellular viable *P*. *acnes* was performed according to previously described methods [14]. For *P*. *acnes* infection, the bacteria were harvested from 3-d cultures and added to the cell cultures (1×10^5^ Raw264.7 cells, Atg5^+/+^ or Atg5^-/-^ MEF cells, and HeLa cells) in a 24-well culture plate at MOI 100 or MOI 1000, without antibiotics. After 1 h, infected cells were washed three times with PBS, and then 10% FBS/DMEM with antibiotics (400 U/ml penicillin G and 1 mg/ml streptomycin) was added to kill the remaining extracellular *P*. *acnes*. The cells were further cultured for the indicated time (postinfection). After the appropriate incubation time, infected Raw264.7 cells were washed three times with PBS and then lysed by dilution in sterile distilled water, whereas infected MEF and HeLa cells were washed three times with PBS and lysed by dilution in sterile distilled water after dispersion by the addition of 0.1 ml of 0.25% trypsin-EDTA (Gibco, Gaithersburg, MD). The number of bacterial colony forming units (CFU) released from the lysed cells was counted after suitable dilutions of the lysates were plated on GAM agar and incubated at 37°C under anaerobic conditions for 5 d. Cell cultures of each sample were performed in triplicate wells and the results for each sample at 24 h, 48 h, and 72 h postinfection are expressed as the rate of infection (% intracellular viable *P*. *acnes*) calculated by dividing the number of bacterial CFU from each well by the mean number of bacterial CFU from triplicate wells at 5 h postinfection.

### Statistical analysis

GraphPAD PRISM ver. 6 (GraphPad Software, Inc, CA, USA) was used for statistical analysis. Student’s *t*-test was used for categorical comparisons between Atg5^+/+^ and Atg5^-/-^ cells in the colony assay. The Kruskal-Wallis test was used for estimating differences between the three cell lines in the colony assays. A *p*-value of less than 0.05 was considered to be statistically significant.

## Results

### *P*. *acnes* in LC3-positive vacuoles

**Fig 1:**
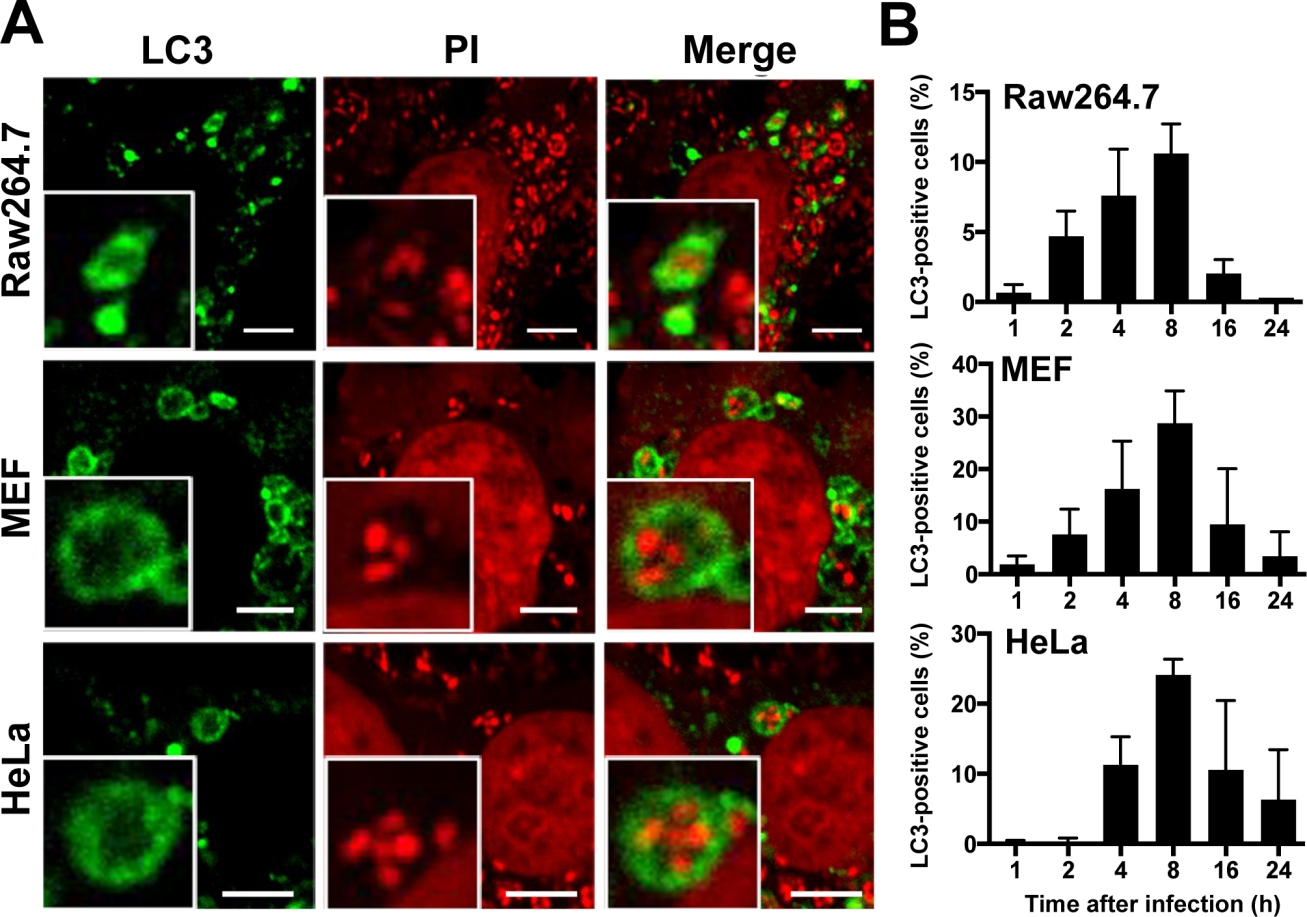
*P*. *acnes* in LC3-positive vacuoles. (A) Confocal microscopic image of LC3-positive vacuoles containing *P*. *acnes* found in Raw264.7, MEF, and HeLa cells at 8 h MOI 1000 postinfection. Scale bar: 5 μm. (B) Frequency of cells with LC3-positive vacuoles containing *P*. *acnes* at 1, 2, 4, 8, 16, and 24 h MOI 1000 postinfection. Data are representative of at least three independent experiments. Black bars; means + SD.

**Fig 2:**
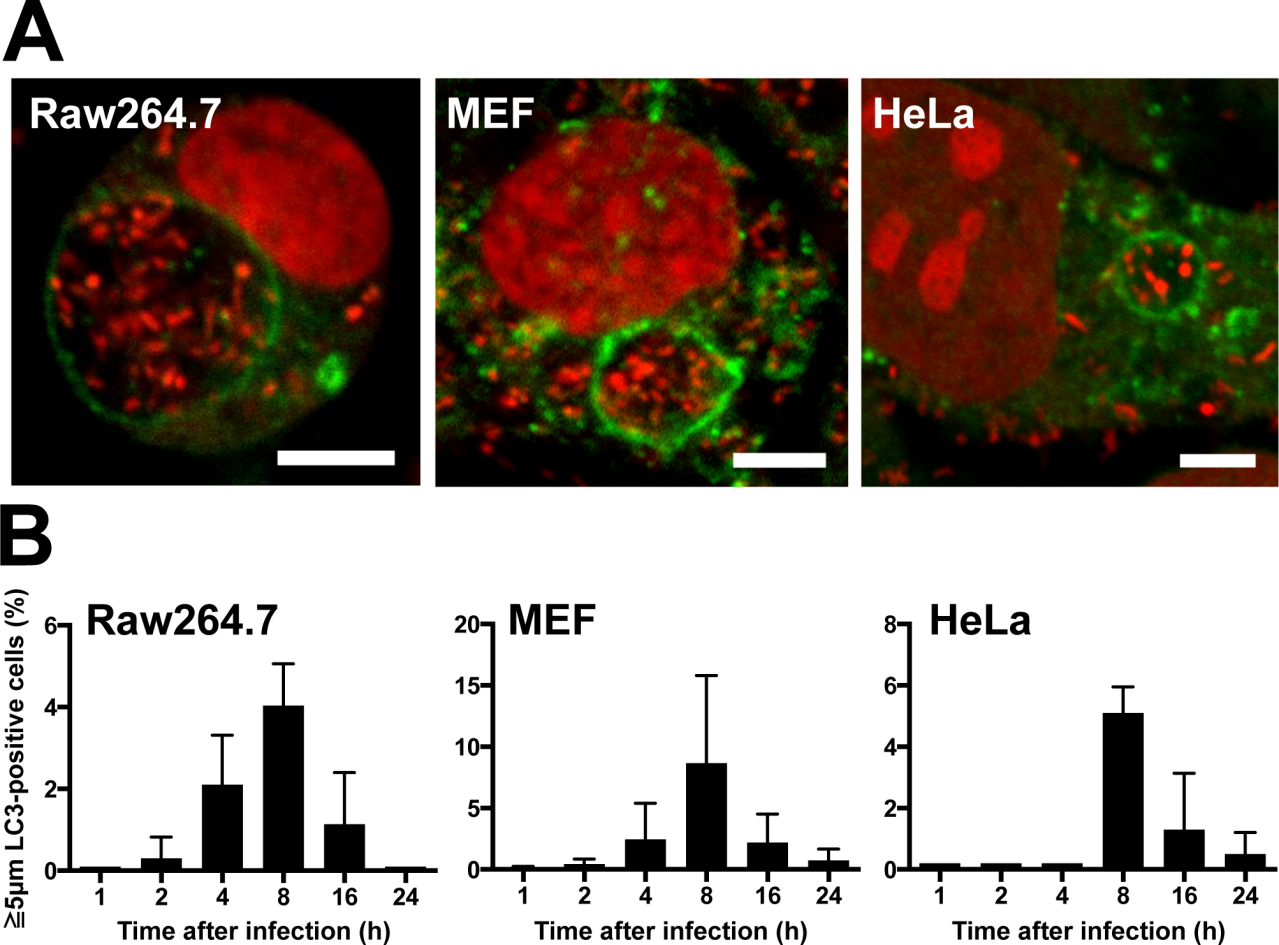
Large LC3-positive vacuoles containing many *P*. *acnes*. (A) Confocal microscopic image of large (≥5 μm in diameter) LC3-positive vacuoles containing many *P*. *acnes* found in Raw264.7, MEF, and HeLa cells at 8 h MOI 1000 postinfection. Scale bar: 5 μm. (B) Frequency of cells with large LC3-positive vacuoles containing *P*. *acnes* at 1, 2, 4, 8, 16, and 24 h MOI 1000 postinfection. Data are representative of at least three independent experiments. Black bars; means + SD.

When Raw264.7, MEF, and HeLa cells were infected with *P*. *acnes* at an MOI of 1000 and examined at 1, 2, 4, 8, 16, and 24 h after infection, *P*. *acnes* were found within the autophagy marker LC3-positive vacuoles from 2 h to 24 h postinfection in all three cell lines. (Fig 1A). LC3-positive vacuoles containing *P*. *acnes* (LC3-positive *P*. *acnes*) were usually less than 5 μm in diameter and contained one or a few *P*. *acnes* cells. The frequency of cells containing LC3-positive *P*. *acnes* was highest at 8 h postinfection in all three cell lines; 11% in Raw264.7, 29% in MEF, and 24% in HeLa cells (Fig 1B). A large LC3-positive vacuole, 5 μm or greater in diameter, containing many *P*. *acnes* cells (Fig 2A) was sometimes observed in all three cell lines, with a peak frequency of 4% in Raw264.7, 9% in MEF, and 5% in HeLa cells, at 8 h postinfection (Fig 2B).

### Requirement for successful induction of LC3-positive *P*. *acnes*

**Fig 3:**
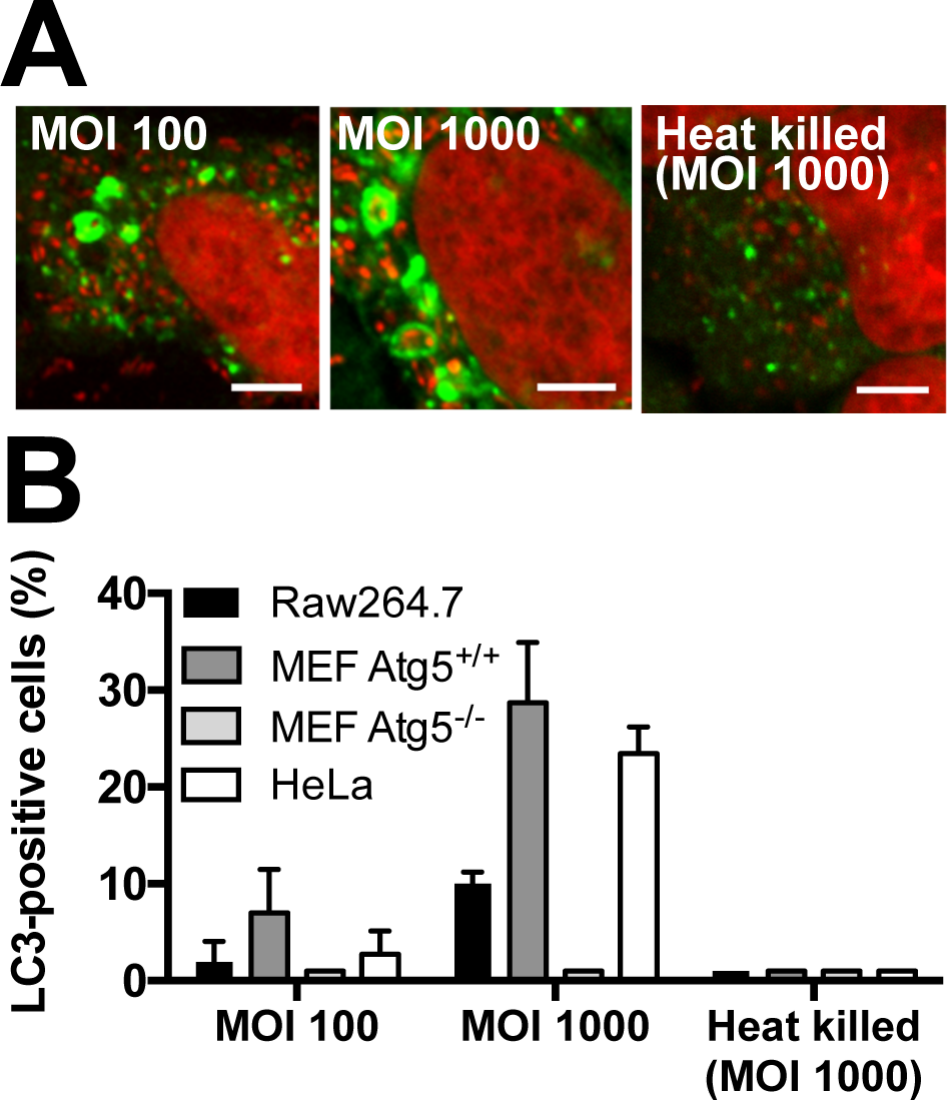
Induction of LC3-positive *P*. *acnes* in different conditions. (A) Confocal microscopic images of *P*. *acnes*-infected HeLa cells at 8 h postinfection. HeLa cells were infected by viable *P*. *acnes* at MOI 100 and 1000 or by heat-killed *P*. *acnes* at MOI 1000. Scale bar: 5 μm. (B) Frequency of cells with LC3-positive vacuoles containing *P*. *acnes* at 8 h postinfection. Raw264.7, MEF (including Atg5^-/-^ cells) and HeLa cells were infected by viable *P*. *acnes* at MOI 100 and 1000 or by heat-killed *P*. *acnes* at MOI 1000. Data are representative of at least three independent experiments. Black bars; means + SD.

The frequency of LC3-positive *P*. *acnes* in Raw264.7, Atg5^+/+^ MEF, Atg5^-/-^ MEF, and HeLa cells at 8 h postinfection was compared in the same experiment among different conditions of *P*. *acnes* used for infection (Fig 3). LC3-positive *P*. *acnes* was never observed under any conditions in autophagy-deficient Atg5^-/-^ MEF cells. In the other three cell lines at MOI 100 of viable *P*. *acnes*, no large LC3-positive vacuoles containing many *P*. *acnes* were found, and the number of small LC3-positive vacuoles containing one or a few *P*. *acnes* was decreased. In all three cell lines, LC3-positive *P*. *acnes* was completely abolished at MOI 1000 of heat-killed *P*. *acnes*, although many *P*. *acnes* cells were found in the cells.

### Contribution of autophagy to intracellular *P*. *acnes* killing

**Fig 4:**
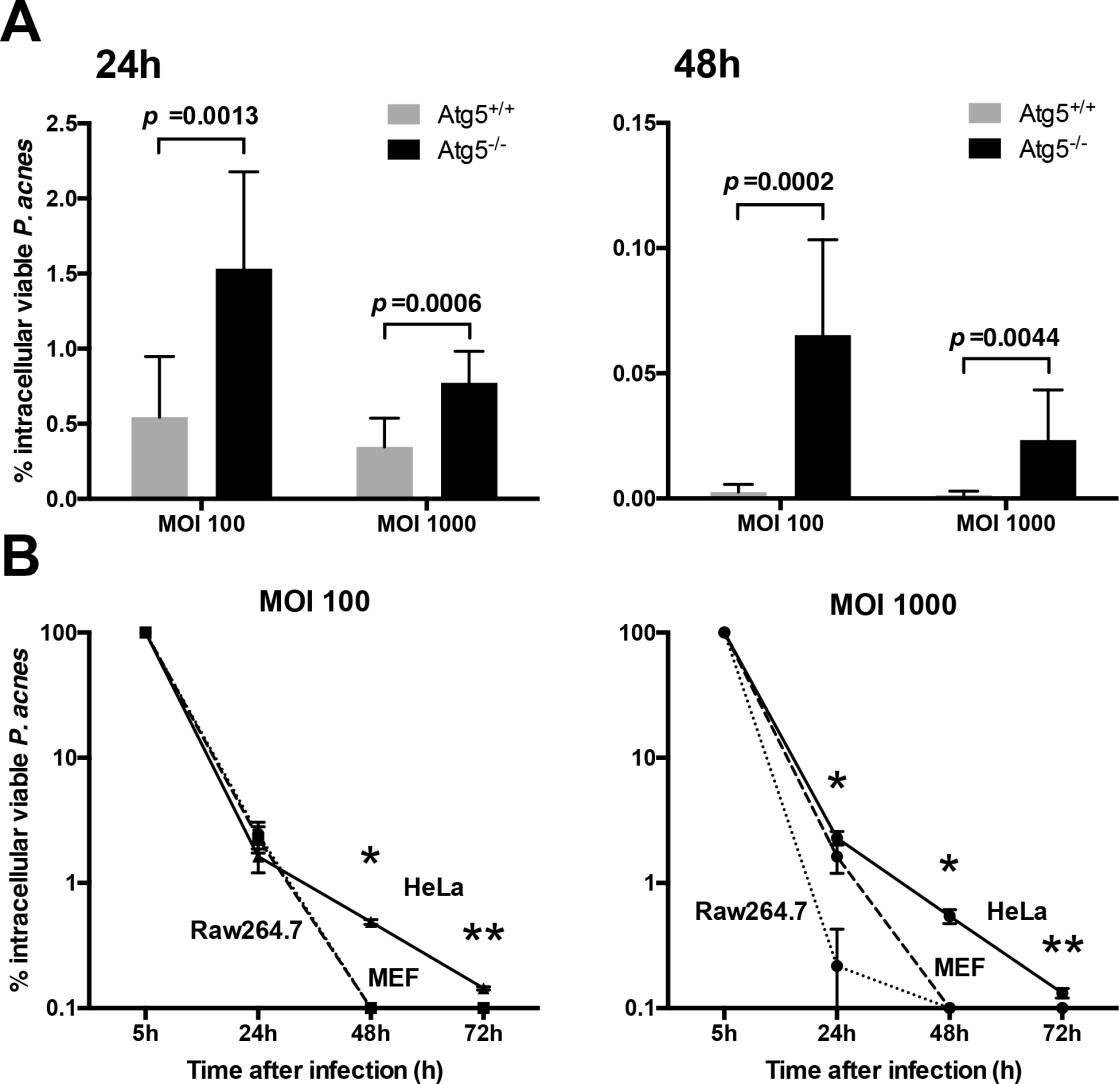
Colony assay for intracellular viable *P*. *acnes*. (A) Autophagy-deficiency allowed for survival and persistence of *P*. *acnes* within the host cells. Both Atg5^+/+^ and Atg5^-/-^ MEF cells were infected by *P*. *acnes* at MOI 100 or 1000. The rate of infection (% intracellular viable *P*. *acnes*) was measured by colony assay at 5, 24, and 48 h postinfection. Black bars; means + SD, n = 9. Data were collected from three independent experiments. (B) Raw264.7, MEF, and HeLa cells were infected by *P*. *acnes* at MOI 100 or 1000. The rate of infection (% intracellular viable *P*. *acnes*) was measured by colony assay at 5, 24, 48, and 72 h postinfection. Black bars; means ± SD, n = 3. *P = 0.0036, **P = 0.036 compared among three cell lines.

The contribution of autophagy to intracellular *P*. *acnes* killing was examined with autophagy-deficient MEF cells. After *P*. *acnes* infection (at MOI 100 or 1000) to MEF (Atg5^+/+^) cells and autophagy-deficient MEF (Atg5^-/-^) cells, the number of intracellular viable *P*. *acnes* was measured by colony assay at 5, 24, and 48 h postinfection (Fig 4A). The rate of infection (% intracellular viable *P*. *acnes*) was 2.8 and 2.2 times higher at 24 h, and 25.3 and 17.6 times higher at 48 h in the Atg5^-/-^ than Atg5^+/+^ MEF cells at MOI 100 and 1000, respectively. LC3-positive *P*. *acnes* was not found in autophagy-deficient Atg5^-/-^ cells at 24 and 48 h after *P*. *acnes* infection at an MOI of 100 or 1000.

### Survival or persistence of intracellular *P*. *acnes*

The rate of infection (% intracellular viable *P*. *acnes*) in Raw264.7, Atg5^+/+^ MEF, and HeLa cells was compared in the same experiment by colony assay at 5, 24, 48, and 72 h after *P*. *acnes* infection at an MOI 100 or 1000 (Fig 4B). The measurement was mostly unsuccessful after 3 d postinfection due to overgrowth of the cultured cells. The mean number (×10^5^ CFU/well) of intracellular viable *P*. *acnes* at 5 h postinfection was 6.8 and 13 in Raw264.7 cells, 4.5 and 15 in MEF cells, and 2.7 and 13 in HeLa cells at MOI 100 and 1000, respectively. These intracellular viable *P*. *acnes* were gradually killed by intracellular digestion and most *P*. *acnes* were killed in Raw264.7 and MEF cells by 48 h postinfection (MOI 100 and 1000). In HeLa cells, a few *P*. *acnes* cells survived at 48 h postinfection and even at 72 h postinfection (mean rate of infection at 72 h in three experiments; 0.18% and 0.14% at MOI 100 and 1000, respectively), although intracellular viable *P*. *acnes* in Raw264.7 and MEF cells were totally abolished by this time.

### Morphology of intracellular *P*. *acnes*

**Fig 5:**
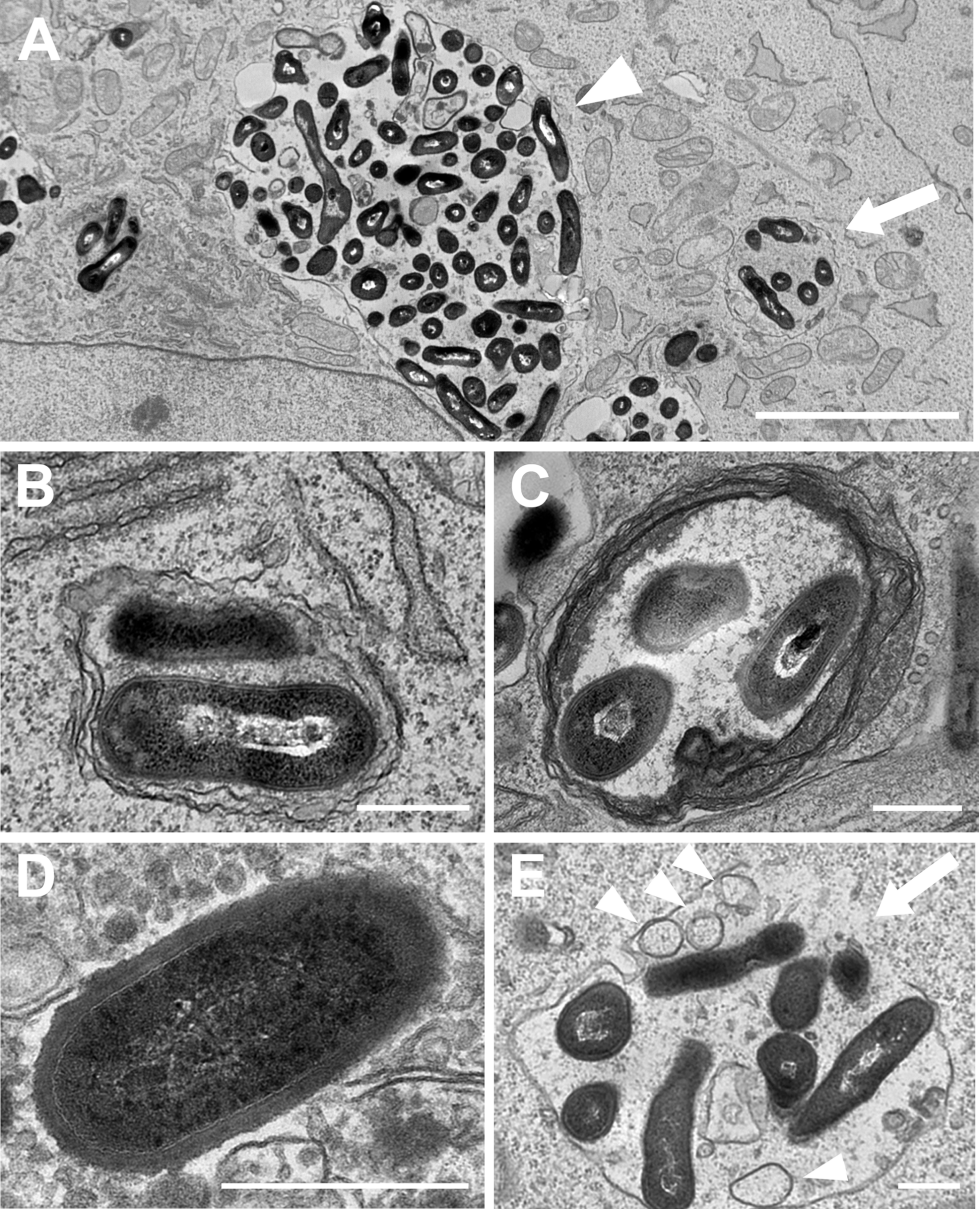
Electron microscopic images of intracellular *P*. *acnes*. HeLa cells infected by *P*. *acnes* at MOI 1000 were examined at 8 h postinfection. (A) Small (indicated by an arrow) and large (indicated by arrowhead) vacuoles in a single HeLa cell, containing a few and many undigested *P*. *acnes* cells, respectively, both surrounded by a single membrane. (B) Two undigested *P*. *acnes* cells interfaced with cytosolic components were surrounded by a double-membrane. (C) Three undigested *P*. *acnes* cells without cytosolic components were surrounded by a multi-layered membrane. (D) A single undigested *P*. *acnes* cell free in cytoplasmic space without a surrounding membrane structure. (E) A late endosome surrounded by a single-membrane with multi-vesicular bodies (arrowheads) containing undigested *P*. *acnes* cells. A part of the single-membrane is discontinued (indicated by an arrow) with a small influx of cytosolic components into the endosome. Scale bar: A 5 μm, B-E 500 nm.

Electron microscopic examination with HeLa cells at 8 h postinfection (MOI 1000) revealed a variety of membrane structures containing digested and undigested *P*. *acnes* cells. Morphologically undigested or digested *P*. *acnes* cells surrounded by a single membrane were most frequently observed. Most of the large vesicles containing many *P*. *acnes* had a single membrane (Fig 5A). Morphologically undigested *P*. *acnes* cells were occasionally surrounded by a double membrane (Fig 5B) or multi-layered membrane (Fig 5C), and sporadically existed free in the cytoplasmic space without a surrounding membrane structure (Fig 5D). Ruptured late-endosomal *P*. *acnes* was identified as having multivesicular bodies, which are characteristic features of the late endosome, and a partially-discontinued single membrane with a small influx of cytosolic components into the endosome (Fig 5E).

### LAMP1 expression in LC3-positive *P*. *acnes*

**Fig 6:**
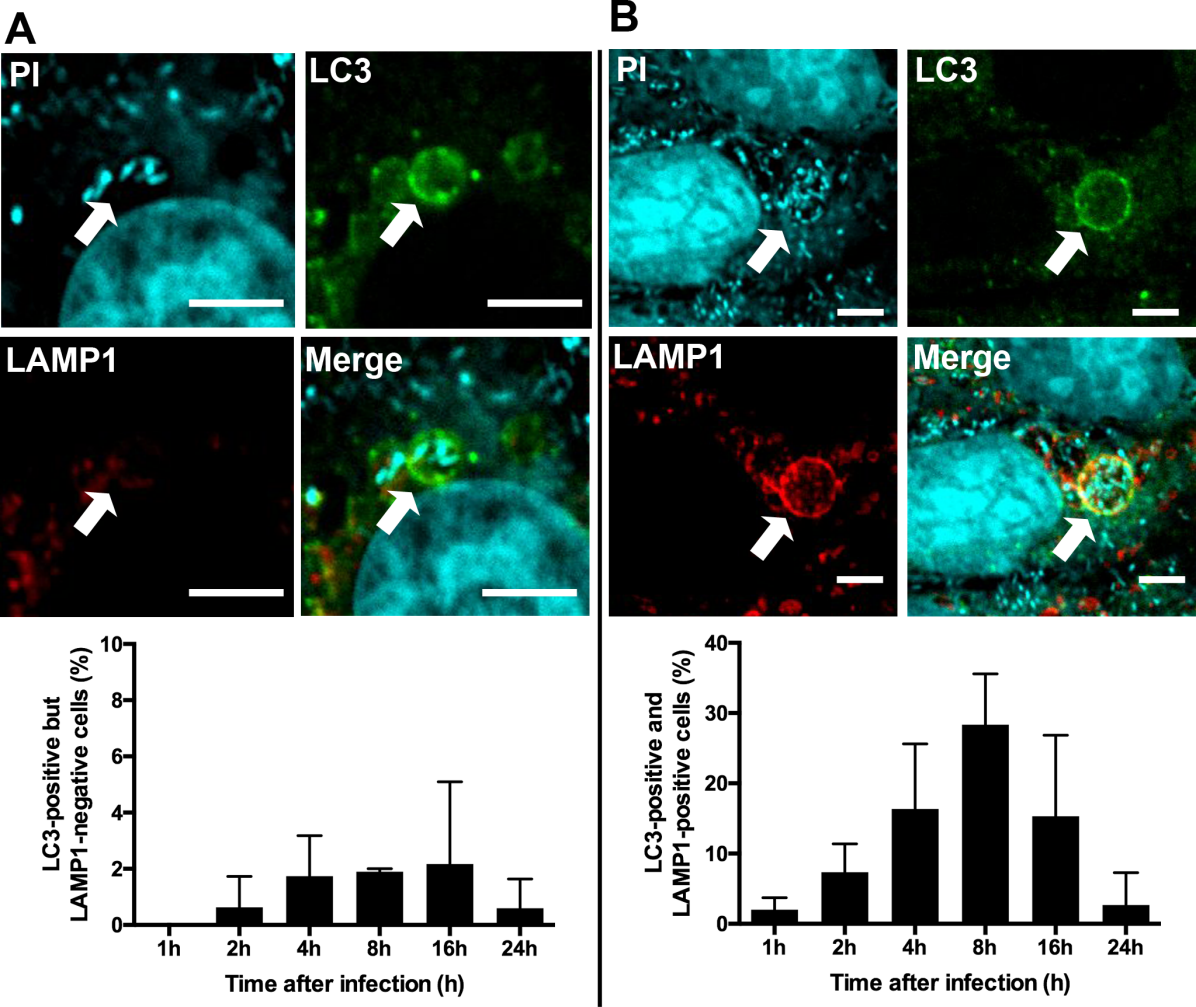
LAMP1 expression of LC3-positive *P*. *acnes*. Confocal images of *P*. *acnes*-infected MEF cells at 8 h MOI 1000 postinfection with fluorescent labeling of LC3 (green), LAMP1 (red), and *P*. *acnes* cells (cyan). (A) A small LC3-positive, but LAMP1-negative, vacuole containing *P*. *acnes* is indicated by an arrow, and the frequency of cells with such vacuoles is shown in the lower graph. (B) A large LC3-positive/LAMP1-positive vacuole is indicated by an arrow, and the frequency of cells with such vacuoles is shown in the lower graph. Scale bar: 5 μm.

Although a few small LC3-positive *P*. *acnes*-containing vacuoles were negative for LAMP1 (Fig 6A), most LC3-positive *P*. *acnes*-containing vacuoles including large vacuoles were also positive for this phagolysosomal marker in all three cell lines at 8 h postinfection (MOI 1000) (Fig 6B). Although the frequency of cells with at least one LC3-positive/ LAMP1-negative vacuole was generally low (< 2%) in the three cell lines, these cells were consistently observed from 2 h to 24 h postinfection. The frequency of cells with at least one LC3 and LAMP1 double-positive vacuole peaked at 8 h postinfection and significantly decreased thereafter.

## Discussion

The findings of the present study demonstrate that *P*. *acnes* induces autophagy in certain conditions in vitro. Autophagy was induced not only in epithelial and mesenchymal cells, but also in macrophages, by intracellular infection with a cell-invasive strain of *P*. *acnes*. In all cell lines, autophagy induction was successful when viable *P*. *acnes* were used for infection and the frequency of the induced autophagy was increased in the presence of a large number of *P*. *acnes*.

Electron microscopic observations and the results from colony assays, especially with autophagy-deficient MEF cells, suggested that intracellular *P*. *acnes* are basically degraded by an endocytic pathway of the infected host cell, but autophagy is induced to support intracellular clearance of the bacterium, especially when infected *in vitro* by large numbers of the bacterium beyond endocytic capacity. Thus, autophagy observed in the present experiments is thought to have been originally induced as a defense mechanism against *P*. *acnes*.

In the present study, large vacuoles containing many *P*. *acnes* cells were observed postinfection at an MOI of 1000, most of which were LC3 and LAMP1-double positive. LC3-positivity of such a large single-membrane vacuole suggested a partial contribution of mechanisms other than the classical concept of autophagy. Recent observations indicated that LC3 is recruited to other membranes, including single-membrane phagosomes, in a process termed LC3-associated phagocytosis [24]. The LAMP1-positivity of most of the LC3-positive large single-membrane vacuoles suggested that many *P*. *acnes* in such vacuoles are in the process of lysosomal digestion and seemed to hardly contribute to bacterial survival. Further experiments of autophagy induction using interferon gamma may be useful to test the anti-bacterial activity of autophagy against *P*. *acnes*.

Apart from such an interesting observation of the large LC3-positive *P*. *acnes*-containing vacuoles, most of the small LC3-positive *P*. *acnes*-containing vacuoles were created by classical autophagy, based on our electron-microscopic observations of a few *P*. *acnes*-containing small vacuoles with double or multiple-layered membranes, ruptured endosomes containing a few *P*. *acnes* cells, and undigested *P*. *acnes* cells free in the cytoplasmic space. The frequency of cells with LC3-positive vacuoles containing *P*. *acnes* was mostly less than 30% of the infected cells and the number of such vacuoles in a single cell was generally small. Probably because of such infrequent autophagy induction, Western blot analysis with an antibody to LC3 showed an ill-defined increase of the LC3 isoform II/I ratio in *P*. *acnes*-infected cells compared to uninfected cells, and the increase was not so defined compared to that in *Streptococcus pyogenes*-infected cells (data not shown).

In the present study, many LC3-positive *P*. *acnes* vacuoles were positive for LAMP1, but a few LC3-positive *P*. *acnes* vacuoles were negative for LAMP1. Such LC3-positive/ LAMP1-negative vacuoles were mostly small-sized and consistently observed in some infected cells for 24 h postinfection. Intracellular viable *P*. *acnes* was detected at 3 d after infection in HeLa cells, but totally abolished by this time in Raw264.7 and MEF cells. These findings suggest that some *P*. *acnes* persist in autophagic vacuoles without further digestion, especially in epithelial cells, in a similar way to *Brucella melitensis* or *Legionella pneumophila* [25,26].

Fischer et al. reported the intracellular persistency of *P*. *acnes* [16]. In that study, a cell-invasive strain of *P*. *acnes* isolated from prostate was used to infect a macrophage cell line derived from human monocytes (THP-1). By colony assay, they detected many viable *P*. *acnes* even at 3 d after infection. While they did not investigate autophagy, they suggested that the intracellular persistence of *P*. *acnes* is caused by cytosolic escape of *P*. *acnes* or intraphagosomal pH neutralization. Whether some cytosolic *P*. *acnes* cells remain free from targeting by autophagy and thus persist in the cells is not clear from the present study. In the present study, intracellular viable *P*. *acnes* was not detected at 3 d after infection in a mouse macrophage cell line, Raw264.4 cells. The different results may be due to differences in the macrophage cell line used for each experiment.

The strain of *P*. *acnes* used for the study was a cell-invasive serotype I isolate from a sarcoid lymph node sample. Our preliminary data suggested a wide variety of autophagy-inducing capacities among the *P*. *acnes* strains so far examined with 20 strains of cell-invasive *P*. *acnes*. Although the reason remains unknown, different capacities of autophagy induction may be associated with different degrees of CAMP factor expression from *P*. *acnes* in endosomes, because the hemolytic activity of CAMP factor [27] may cause endosomal rupture. It was originally thought that the bacterium escaped from the ruptured endosome [17,19,28] or the damaged endosome containing the bacterium [29] induces autophagy. The possibility that such endosomal rupture or damage is caused by active secretion of a CAMP factor or other products from viable *P*. *acnes* is supported by our observation that heat-killed (intracellular) *P*. *acnes* did not induce autophagy. According to the report by Furukawa et al. [14], some serotype I *P*. *acnes*, including ATCC 6919, and all serotype II *P*. *acnes*, including ATCC 11828, do not invade epithelial cells. While the non-invasive isolates can induce autophagy in macrophages, they are unable to induce autophagy in MEF and HeLa cells, because they cannot invade the cells to induce intracellular infection.

The cytosolic receptors for bacterial peptidoglycan, NOD1 and NOD2, interact with ATG16L1 and mediate ATG16L1 recruitment to the site of bacterial entry in non-phagocytic cells, macrophages, and lymphocytes [30]. NOD2 triggered by muramyldipeptide induces autophagy in dendritic cells and defects in this pathway due to NOD2 or ATG16L1 risk variants in Crohn’s disease, another granulomatous disease, could result in defective lysosome-mediated destruction and immune-mediated clearance, thus enhancing bacterial persistence [31]. Tanabe et al. reported that intracellular *P*. *acnes* activated NF-*κ*B in both an NOD1- and NOD2-dependent manner [15]. It is likely that some *P*. *acnes* cells escape from endosomes after intracellular infection and activate cytosolic NOD receptors, leading to autophagy induction. An investigation of NOD1 gene polymorphisms in Japanese sarcoidosis patients suggested the involvement of the NOD1 796A-allele, which is linked to reduced NOD1 expression and decreased NF-*κ*B activation due to intracellular *P*. *acnes* [15]. Reduced NOD1 expression in sarcoidosis patients may impair autophagy induction and affect the supportive role of autophagy for intracellular bacterial killing, leading to enhanced intracellular survival and persistence of *P*. *acnes*. To address this possibility, these same experiments should be performed using human macrophages and epithelial cells from subjects with or without the NOD1 796A-allele.

Negi et al. reported a higher frequency of latent *P*. *acnes* infection in sinus macrophages of the lymph nodes from sarcoidosis patients than from non-sarcoidosis patients by immunohistochemistry with *P*. *acnes*-specific monoclonal antibodies [9]. They also demonstrated many *P*. *acnes* in some macrophages and granuloma cells at the site of sarcoid granulomatous inflammation, suggesting intracellular proliferation of persistent *P*. *acnes*. Eishi [32] proposed three critical conditions required for the development of *P*. *acnes*-induced sarcoidosis: (1) latent *P*. *acnes* infection, (2) activation of persistent *P*. *acnes* triggered by particular environmental factors, and (3) a hypersensitive Th1 immune response to the proliferation of intracellular *P*. *acnes*. The findings of the present study suggest that latent *P*. *acnes* infection could occur even in healthy patients or in non-sarcoidosis patients by a conventional autophagy process in epithelial cells. Indeed, latent *P*. *acnes* infection has been demonstrated in glandular epithelial cells of the prostate from non-sarcoidosis patients with prostate cancer or bladder cancer [33]. Although intracellular persistence of *P*. *acnes* in macrophages could not be demonstrated in the present study, possible impairment of autophagy induction in many sarcoidosis patients and some non-sarcoidosis subjects with the NOD1 796A-allele may permit latent infection even in macrophages by increasing the cytosolic *P*. *acnes*, which seems to proliferate intracellularly more readily than *P*. *acnes* in autophagic vacuoles.

**Fig 7:**
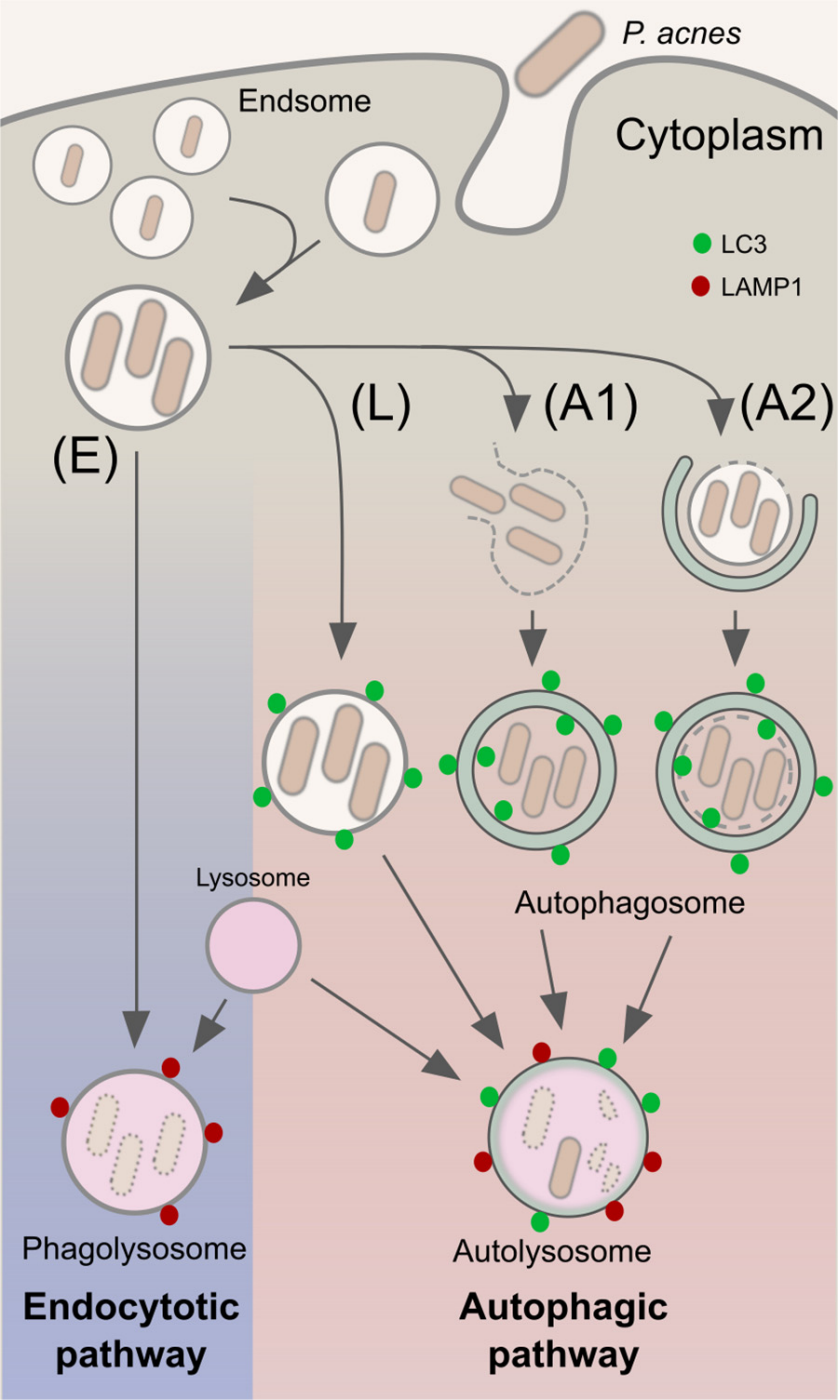
Trafficking pathways of intracellular *P*. *acnes*. Intracellular *P*. *acnes* are digested in both endocytic (E) and autophagic (A) pathways. Autophagy induction is caused by cytosolic *P*. *acnes* cells that completely escaped from endosomes (A1) or by damaged endosomes containing *P*. *acnes* cells (A2). LC3-associated phagocytosis (L) is especially induced in the presence of infection by a large number of bacteria.

The results of the present study suggest two main pathways in the lysosomal degradation of intracellular *P*. *acnes* (Fig 7). *P*. *acnes* are basically degraded by an endocytic pathway after being internalized into host cells via endocytosis or phagocytosis. When endosomes containing the bacteria are damaged or the bacteria completely escape from the damaged endosomes, these damaged endosomes and the cytosolic bacteria are processed in the autophagic pathway by forming multi-layered or double-membrane vacuoles (autophagosomes), respectively. The recently proposed pathway via LC3-associated phagocytosis may also contribute to intracellular degradation by forming single-membrane vacuoles. The frequency of autophagy induction and the size of autophagosomes depend on the number of intracellular bacteria. Finally, *P*. *acnes* are degraded by lysosomal enzymes in LC3 and LAMP1 double-positive autolysosomes. Thus, autophagy induced by intracellular *P*. *acnes* infection contributes to intracellular bacterial killing as an additional host defense mechanism to conventional endocytosis or phagocytosis. Further studies with primary cells such as primary keratinocytes and/or macrophages/neutrophils purified from blood are required to confirm the role of autophagy induced by intracellular *P*. *acnes*.

## Supporting Information

S1 File Data for Fig 1B: Frequency of cells with LC3-positive vacuoles containing *P*. *acnes* in each cell line at 1, 2, 4, 8, 16, and 24 h MOI 1000 postinfection. Data for Fig 2B: Frequency of cells with large LC3-positive vacuoles containing *P*. *acnes* in each cell line at 1, 2, 4, 8, 16, and 24 h MOI 1000 postinfection. Data for Fig 3B: Frequency of cells with LC3-positive vacuoles containing *P*. *acnes* in each cell line at 8 h postinfection. Data for Fig 4A: The rate of infection (% intracellular viable *P*. *acnes*) in Atg5^+/+^ and Atg5^-/-^ MEF cells at MOI 100 or 1000 at 24, and 48 h postinfection, calculated by dividing the number of bacterial CFU from each well by the mean number of bacterial CFU from at 5 h postinfection. Data for Fig 4B: The rate of infection (% intracellular viable *P*. *acnes*) in each cell line at 24, 48, and 72 h postinfection, calculated by dividing the number of bacterial CFU from each well by the mean number of bacterial CFU at 5 h postinfection. Data for Fig 6A: The frequency of a small LC3-positive, but LAMP1-negative, vacuole containing *P*. *acnes* in *P*. *acnes*-infected MEF cells at MOI 1000 for 24 h postinfection. Data for Fig 6B: The frequency of a large LC3-positive/LAMP1-positive vacuole in *P*. *acnes*-infected MEF cells at MOI 1000 for 24 h postinfection.
(XLSX)

## Abbreviations

DMEM: Dulbecco's Modified Eagle Medium
FBS: fetal bovine serum
MHC: major histocompatibility complex
MOI: multiplicity of infection
NF-kB: nuclear factor kappa B
PBS: phosphate buffered saline
Th1: T-cell type 1
